# Three Dimensional Myocardial Deformation and Diagnosis of Stable Coronary Artery Disease

**Published:** 2014-12-19

**Authors:** V Parisi, G Galasso, F Pastore, S De Luca, E Allocca, E Capuano, B Trimarco, N Ferrara

**Affiliations:** 1 Department of Translational Medical Sciences - Federico II University;; 2 Department of Advanced Biomedical Sciences-Federico II University;; 3 Department of Cardiology, AOU “Maggiore Della Carità”, Novara

**Keywords:** *Coronary Artery Disease*, *Echocardiography*, *Three Dimensional Transthoracic Echocardiography*, *Ischemic Heart Disease*

## Abstract

To date, only one third of patients, with stable angina, undergoing coronary angiography demonstrated obstructive coronary artery disease (CAD). Thus, identifying high sensitivity and specificity, low-cost, non invasive tests is crucial. Here we present the case of a patient, at a high risk of CAD, undergoing coronary angiography because of positive exercise test and stress imaging results, with non obstructive coronary artery disease at angiography, confirmed by FFR. Interestingly, 3D speckle tracking, performed before angiography, assessed normal left ventricle deformation, predicting the absence of severe coronary artery lesions.

## I. INTRODUCTION

Patients with suspected coronary artery disease (CAD) are commonly evaluated by non-invasive tests in order to identify those that must be referred to coronary angiography. However, it has been demonstrated that only one third, of patients with positive non invasive testing before coronary angiography have obstructive CAD at catheterization [1]. Therefore, the issue of identifying high sensitivity and specificity non invasive tests is still open and is crucial in the clinical and economical management of patients with stable CAD. Two dimensional (2D) and 3D speckle tracking echocardiography demonstrated ability in detecting subtle myocardial deformation abnormalities in CAD patients with significant coronary artery stenosis, even at rest [2–4]. However, despite numerous data available on 2D speckle tracking echocardiography, its technical limitations limit its use in clinical practice; noteworthy, on 3D speckle tracking, which appear to overcome these limits, still few data are available. Here we present the case of a patient, at a high risk of CAD, undergoing coronary angiography because of positive exercise test and stress imaging results, then diagnosed with vasospastic angina after angiography showing non obstructive coronary artery disease. Interestingly, 3D speckle tracking, performed before angiography, assessed normal left ventricle deformation, predicting the absence of severe coronary artery lesions.

## II. CLINICAL CASE

**Figure 1.**
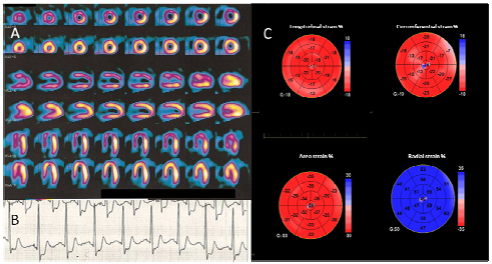
**Perfusional Imaging. A:** Single Photon Emission Computed Tomography showing severe myocardial perfusion abnormalities of the interventricular septum and the apex. **B:** Representative lead (V5) with ST segment depression during exercise stress test. **C:** Three dimensional global and regional longitudinal, circumferential, area and radial strain represented by a bull-eye graph showing no regional deformation abnormalities.

A 46 years old sportive man, non-smoker, with history of hypertension, hyperlipidemia, and positive family history for cardiovascular disease. After an and episode of chest pain during physical activity, lasting few minutes and spontaneously recovered, he underwent to an exercise test which showed inducible ischemia, with ST depression of 4 mm at the V4–V6 leads associated with atypical chest pain. Myocardial perfusion scintigraphy (SPECT) showed severe myocardial perfusion abnormalities of the anteroseptal wall and in the apex with reduced systolic function during stress (Figure 1). He was referred to coronary angiography with diagnosis of stable angina.

**Figure 2.**
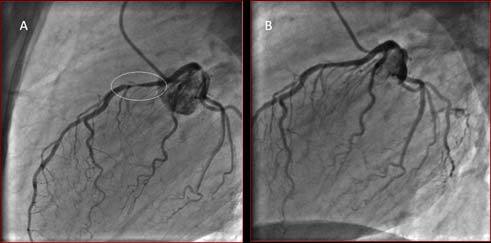
**Coronary Angiography. A:** Coronary angiography showing a severe proximal LAD stenosis (circle). **B:** After 300µg nitroglycerine intracoronary, the stenosis was almost completely resolved, fractional flow reserve performed to evaluate the residual stenosis confirmed the absence of flow limiting stenosis.

At hospital admission the patient had a normal resting electrocardiogram. Laboratory blood tests examinations were in the normal ranges. The echocardiographic examination, performed with a GE Vivid E9 machine, was normal. In particular, there was a normal systolic function of left ventricle with no regional abnormalities (Ejection Fraction 60%). A 3D apical view was acquired for 3D speckle tracking analysis. Longitudinal, circumferential, area and radial strain were measured using the dedicated software package (Echopac, GE Vingmed ultrasound, Horten Norway) and showed no regional deformation abnormalities (Figure 2).

The coronary angiography, showed a stenosis of the left anterior descending artery (LAD). After administration of nitrates, the stenosis was significantly reduced (Figure 2). Then, residual stenosis was tested by fractional flow reserve (FFR) confirming the absence of hemodynamic significance (FFR= 0.96 after 3 intracoronary bolus of 60µg, 120 µg and 240 µg adenosine). The patient was then discharged with diagnosis of vasospastic angina.

## III. DISCUSSION

The sensitivity of strain has made it a very effective tool in the evaluation of subclinical heart disease [5]. However, even if we have several years of literature exploring clinical applicability of strain, with encouraging results, strain measure obtained by Doppler velocities and 2D speckle tracking techniques showed important limitation that not allowed its use in clinical practice. Three dimensional speckle-tracking imaging represents a new developed technique overcoming the limitation of 2D out-of-plane speckle motion [6]. However, only few studies explored the ability of this technique in studying regional and global myocardial function in presence or in suspicious of coronary artery stenosis. Indeed, current methods in assessing ischemic risk present several limitations, especially in particular settings. Coronary artery spasm is an important functional abnormality playing a significant role in the pathogenesis of a wide variety of ischemic heart disease [7]. The diagnosis of vasospastic angina is often made by evidence of severe chest pain, usually without physical effort and with a concurrent ECG showing transient ST elevation [8]. However, coronary spasm develops transiently, and, consequently, it is not so easy to document spontaneous attack in the clinical situation. Exercise testing may be helpful, although approximately equal numbers of patients show ST depression, ST elevation, or no change during the exercise [9].

This clinical case highlights the utility of new developed technology in the study of regional myocardial deformation, especially in particular settings, such as stable patients with atypical presentation. In particular, in the presented case, 3D speckle tracking showing no deformational abnormalities, was the only technique, ruling out the presence of severe coronary artery stenosis. Indeed, clinical symptoms, such as effort angina, ECG exercise changes, and a significant perfusion defect showed by SPECT were all supporting the hypothesis of left descending coronary artery severe lesion. According to the published guidelines on management of stable angina [10] the described patient had a high probability of coronary disease (91%), supporting the physician indication to coronary angiography. Coronary angiography holds a fundamental position in the investigation of patients with stable angina, providing reliable anatomical information to identify the presence or absence of coronary lumen stenosis and define therapeutic options. However, the composite rate of major complications associated with routine diagnostic catheterization in stable patients is between 1 and 2%. Consequently, it is a relevant issue to identify noninvasive tests able to improve the appropriateness of coronary angiography. Interestingly, in our case, 3D speckle tracking showed normal and uniform left ventricular deformation (Figure 1), where, in presence of severe CAD, regional abnormalities related to the coronary artery territory distribution should be expected, due to chronic myocardial hypoperfusion. Considering the demonstrated ability of left ventricular deformational analysis in detecting ischemic myocardial regions, 3D speckle tracking may be considered as an additive diagnostic tool supporting the physician in the decision making process of stable angina, helping to identify patients with misleading results at non invasive tests.
